# Supplementary material for: Multi-view information fusion using multi-view variational autoencoder to predict proximal femoral fracture load
Source: Front Endocrinol (Lausanne). 2023 Nov 21;14:1261088. doi: 10.3389/fendo.2023.1261088 (PMC10710145; doi:10.3389/fendo.2023.1261088)
Supplement: Supplementary file 1 [file DataSheet_1.docx]

Supplementary Material

# Supplementary Data

**Table S1.** DXA-derived imaging features. LEAN: grams of lean tissue; FAT: grams of fat tissue; PFAT: percentage of fat tissue; BMD: bone mineral density in gram/cm^2^; BMC: bone mineral content in gram; MASS, mass in grams of the corresponding region; AREA: area of the corresponding region in cm^2^; VOLUME: volume of the corresponding region in cm^3^.

| Feature name | Index | Description |
| --- | --- | --- |
| ANDROID_GYNOID_RATIO | 1 | android-gynoid percent fat ratio |
| ANDROID_FAT/LEAN/MASS/PFAT | 2-5 | android bone |
| APPENDAGE_LEAN_MASS_HEIGHT_2 | 6 | appendage lean mass/height^2^ |
| APPENDAGE_PURE_LEAN_HEIGHT_2 | 7 | appendage pure lean mass/height^2^ |
| ARM_LENGTH | 8 | length of arm |
| BODY_MASS_INDEX | 9 | body mass index |
| BODY_WIDTH | 10 | body width in cm |
| CAVITY_WIDTH | 11 | cavity width in cm |
| FAT_MASS_HEIGHT_SQUARED | 12 | FMI, a measure of relative fat content |
| FAT_MASS_RATIO | 13 | Men: 64 – (20 x height/waist circumference) = RFM |
| GYNOID_FAT/LEAN/MASS/PFAT | 14-17 | gynoid bone |
| HEAD_AREA/BMC/BMD/FAT/LEAN/MASS/PFAT | 18-24 | femur head |
| HTOT_AREA/BMC/BMD | 25-27 | total hip |
| INTER_AREA/BMC/BMD | 28-30 | inter-trochanter bone |
| L1/L2/L3/L4/L5_AREA/BMC/BMD | 31-44 | L1/L2/L3/L4/L5 bone from spine |
| LARM_AREA/BMC/BMD/FAT/LEAN/MASS/PFAT | 45-51 | left arm region |
| LEAN_MASS_HEIGHT_SQUARED | 52 | lean mass/height2 |
| LLEG_AREA/BMC/BMD/FAT/LEAN/MASS/PFAT | 53-59 | bone of left leg |
| LRIB_AREA/BMC/BMD | 60-62 | bones of left rib |
| L_S_AREA/BMC/BMD | 63-65 |  |
| NECK_AREA/BMC/BMD | 66-68 | bone of femur neck |
| OUTER_WALL_WIDTH | 69 |  |
| PELV_AREA/BMC/BMD | 70-72 |  |
| PURE_LEAN_HEIGHT_SQUARED | 73 |  |
| RARM_AREA/BMC/BMD/FAT/LEAN/MASS/PFAT | 74-80 | bone of right arm |
| RLEG_AREA/BMC/BMD | 81-83 | bone of right leg |
| ROI_HEIGHT/TYPE/WIDTH | 84-86 | forearm region of interest |
| RRIB_AREA/BMC/BMD | 87-89 | bones of right rib |
| RTOT_AREA/BMC/BMD | 90-92 | bone of total radius |
| RU13TOT_AREA/BMC/BMD | 93-95 | bone of 1/3 distal radius and ulna |
| RUMIDTOT_AREA/BMC/BMD | 96-98 | bone of mid distal radius and ulna |
| RUTOT_AREA/BMC/BMD | 99-101 | bone of radius and ulna |
| RUUDTOT_AREA/BMC/BMD | 102-104 | bone of ultra distal radius and ulna |
| R_13_AREA/BMC/BMD | 105-107 | bone of 1/3 distal radius |
| R_LEG_FAT/LEAN/MASS/PFAT | 108-111 | bone of right leg |
| R_MID_AREA/BMC/BMD | 112-114 | bone of mid distal radius |
| R_UD_AREA/BMC/BMD | 115-117 | bone of ultra distal radius bone |
| SAT_AREA/MASS/VOLUME | 118-120 | subcutaneous adipose tissue |
| SUBCU_FAT_CORRECTION | 121 | correction for subcutaneous fat |
| SUBTOT_AREA/BMC/BMD/FAT/LEAN/MASS/PFAT | 122-128 | total bone area |
| TAT_AREA/MASS/VOLUME | 129-131 | total adipose tissue |
| TBS L1/L2/L3/L4/L1-L4/L2-L4 | 132-137 | trabecular bone score |
| TOTAL_FAT/FAT_MASS/LEAN/LEAN_MASS/MASS/PFAT | 138-144 | bones of total android |
| TOT_AREA/BMC/BMD | 145-147 | bones of total spine |
| TORCH_AREA/BMC/BMD | 148-150 | bone of trochanter |
| TRUNK_LIMB_FAT_MASS_RATIO | 151 | trunk/limb fat mass  ratio of fat |
| TRUNK_FAT/LEAN/MASS/PFAT | 152-155 | bones of the trunk |
| T_S_AREA/BMC/BMD | 156-158 | bone of thoracic region. |
| UTOT_AREA/BMC/BMD | 159-161 | bone of total ulna |
| U_13_AREA/BMC/BMD | 162-164 | bone of 1/3 distal ulna |
| U_MID_AREA/BMC/BMD | 165-167 | bone of mid distal ulna |
| U_UD_AREA/BMC/BMD | 168-170 | bone of ultra distal ulna |
| VFAT_BODY_FAT/LEAN/MASS/PFAT | 171-174 | visceral |
| VFAT_CAVITY_FAT/LEAN/MASS/PFAT | 175-178 | visceral adipose tissue of abdominal cavity |
| VFAT_OUTERWALL_FAT/LEAN/MASS/PFAT | 179-182 | visceral adipose tissue of abdominal wall |
| VFAT_VOLUME/MASS/AREA | 183-185 | visceral adipose tissue of abdominal volume |
| WAIST_CIRCUMFERENCE | 186 | circumference of waist |
| WARDS_AREA/BMC/BMD | 187-189 | ward's triangle bone |
| WBTOT_AREA/BMC/BMD/FAT/LEAN/MASS/PFAT | 190-196 | whole body |

**2. Histograms of the demographic information**


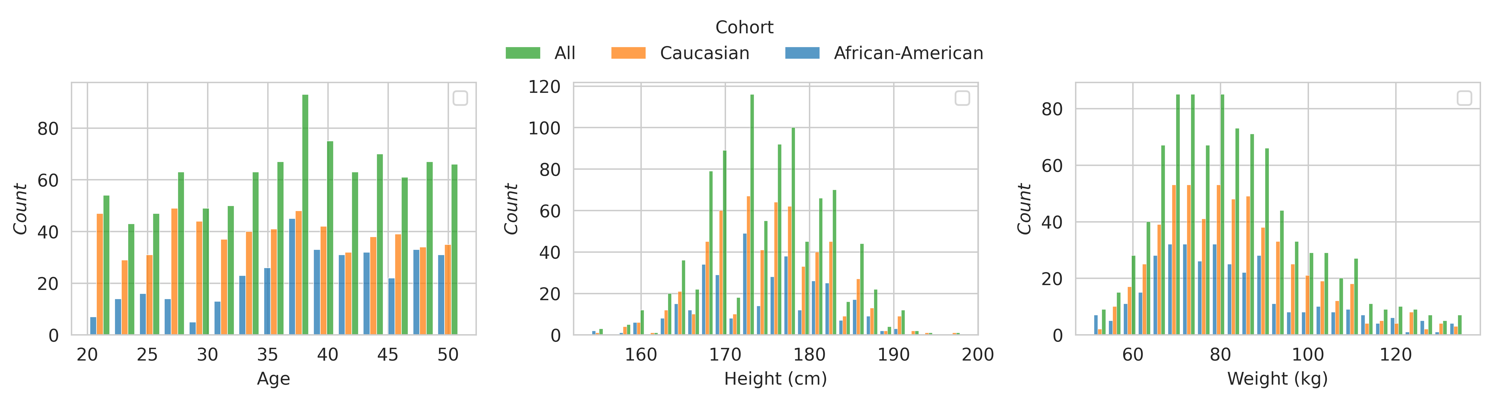


Figure S1. Histograms of the demographic information for the enrolled subjects.

**3. Histograms of the proximal femoral strengths under three loading conditions.**


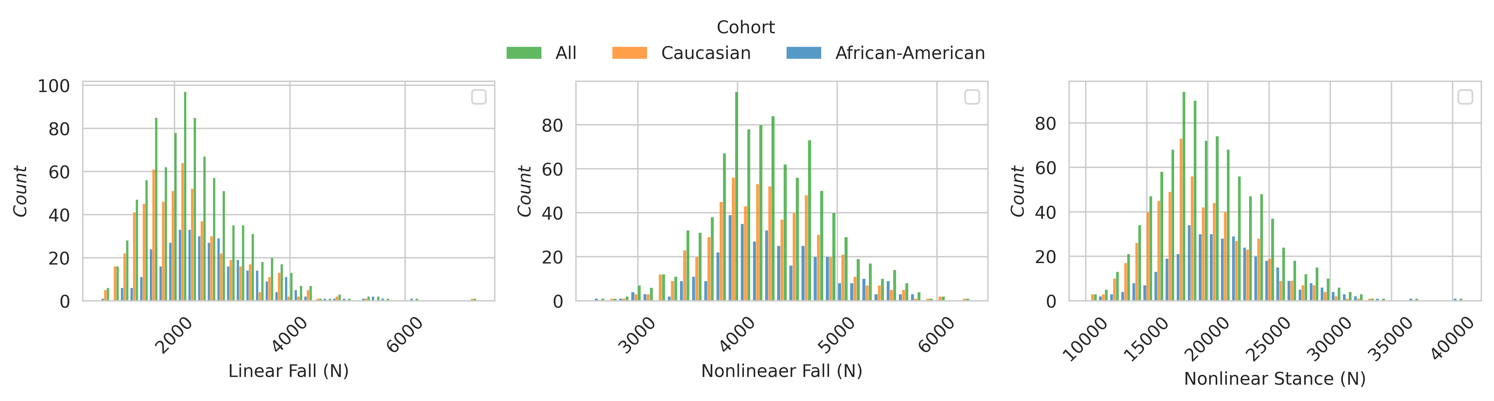
Figure S2. Histograms of the proximal femoral strengths under three loading conditions for the enrolled subjects.

4. Inclusion and exclusion criteria for LOS study

Individuals must meet the following inclusion criteria to be eligible to participate in the study: 1) ≥ 18 years of age (to ensure peak bone mass is attained), 2) Caucasian, African American, or Asian, 3) willing to participate in the study and attend exam for bone densitometry and blood draw; and 4) can speak and understand spoken English.

We will adopt the following exclusion criteria to minimize nongenetic influence on bone mass variation so as to empirically enhance the importance of individual genetic factors for bone mass. These criteria will be assessed based on the subject’s answers.

1. Female subjects who are or could be pregnant;
2. Female subjects who have had bilateral oophorectomy;
3. Serious residuals from cerebral vascular disease;
4. Diabetes mellitus, except for those controlled under medication;
5. Chronic renal failure;
6. Chronic liver failure;
7. Significant chronic lung disease;
8. Alcohol abuse as defined by those who cannot limit drinking, get drunk regularly, and cannot fulfill major responsibilities at work, school, or home;
9. Chronic obstructive pulmonary disease (COPD);
10. Corticosteroid therapy at pharmacologic levels for more than 6 months duration;
11. Treatment with anticonvulsant therapy for more than 6 months duration;
12. Evidence of other metabolic or inherited bone disease such as hyper- or hypoparathyroidism, Paget's disease, osteomalacia, osteogenesis imperfecta or others;
13. Rheumatoid arthritis (except for minor cases that involve only hand joint and wrist);
14. Collagen disease (i.e., osteogenesis imperfecta and hypochondrogenesis);
15. Chronic gastrointestinal diseases including celiac disease, post gastrectomy, Crohn’s disease, ulcerative colitis, liver transplant, cirrhosis;
16. Upper or lower limb loss or disability;
17. HIV infection.

Since our dual-energy X-ray absorptiometry (DXA) machine cannot operate if the subject’s body weight is over 300 pounds, we will not recruit a subject whose body weight is over 300 pounds.
